# Beyond Iron Supplementation: A Narrative Review of the Structural Determinants of Anaemia in Pregnancy in India

**DOI:** 10.7759/cureus.116541

**Published:** 2026-09-19

**Authors:** Sarasa Sahoo, Madhumanti Sarkar, Pinky Jena

**Affiliations:** 1 Anesthesia and Critical Care, Pandit Dindayal Upadhyay Medical College, Rajkot, IND; 2 Obstetrics and Gynaecology, All India Institute of Medical Sciences Rajkot, Rajkot, IND

**Keywords:** anaemia in pregnancy - india, anaemia mukt bharat, haemoglobinopathy, iron deficiency, maternal health, national family health survey, social determinants of health

## Abstract

India's anaemia burden is not a single problem but at least four overlapping ones. The first is uncertainty in burden assessment, illustrated by substantially different capillary versus venous haemoglobin estimates and the removal of haemoglobin testing from the sixth National Family Health Survey (NFHS-6). The second is a biomedical framing miscalculation, in which iron deficiency anaemia is treated as synonymous with anaemia despite evidence that iron deficiency accounts for only about half of cases. The third is a health system design problem, reflected in repeated strategic overhauls of Anaemia Mukt Bharat itself. The fourth is a deep-seated sociostructural problem rooted in teenage marriage, intra-household food allocation, limited female autonomy, gender inequality, and cumulative iron deficits carried over from adolescence and from closely spaced prior pregnancies.

We compared India's experience with selected anaemia-control approaches in Sri Lanka, Bangladesh, and the Philippines, which paired supplementation with food systems and empowerment-based interventions.

We conclude that a supplementation-first model cannot resolve this burden, however robustly financed, and propose an approach that restores methodologically defensible surveillance, differentiates aetiology at the point of care, intervenes before conception rather than only at antenatal registration, and makes cross-sectoral governance enforceable. In the comparator countries, staple-food fortification, delivery across the reproductive lifecycle, and community engagement of households were central to their lower prevalence.

## Introduction and background

Anaemia in pregnancy is a condition with well-characterised pathophysiology, yet its causation in the Indian context is multifactorial and extends well beyond what iron and folic acid supplementation alone can address. India has run a national anaemia control programme since the 1970s, most recently as Anaemia Mukt Bharat (AMB), with revised operational guidelines issued in 2026, yet anaemia in pregnancy remains highly prevalent [1,2]. According to the Fifth National Family Health Survey (NFHS-5, 2019-21), just over half of pregnant women in India were anaemic, though this figure is sensitive to the diagnostic cut-off applied [1,3]. A recent synthesis of Indian cohort data has linked maternal anaemia to adverse pregnancy, delivery, and neonatal outcomes [4].

The persistence of this burden despite sustained investment is difficult to explain. It cannot be attributed to a lack of political attention: anaemia reduction has been a named target of the POSHAN Abhiyaan national nutrition mission, has its own dashboard and digital monitoring infrastructure, and underwent a second major strategic redesign in 2026 [2,5]. Nor can it be attributed simply to the non-availability of iron supplements, which are distributed to most pregnant women, although distribution alone does not guarantee adequate quantity, uninterrupted availability, or adherence. The clinical significance of anaemia extends beyond maternal morbidity. It is implicated, either as a direct cause or as a contributing factor, in an estimated 18-20% of maternal deaths in India [6].

This review advances the argument that India's persistently high anaemia burden in pregnancy cannot be adequately explained by a failure of medical knowledge, drug availability, or even political will. Instead, it reflects a structural mismatch between a largely biomedical, supplementation-centred programme architecture and a problem whose determinants extend substantially beyond clinical supplementation.

These determinants span: 1. epidemiological measurement uncertainty, 2. multi-aetiological biology, 3. health-system design, and 4. deeply embedded social and gender structures that operate long before a woman ever enters an antenatal clinic. In this review, we examine each of these four domains and situate India's trajectory against comparator countries that have achieved larger declines.

## Review

Literature search and selection

This is a narrative rather than a systematic review. The disciplines relevant to the question include: i) epidemiology, ii) clinical haematology, iii) health systems research and demography, and iv) gender studies. This review was therefore designed as a narrative synthesis rather than a systematic review, drawing purposively on evidence across these disciplines. We searched PubMed, Google Scholar, and specialty journals (including the Indian Journal of Haematology and Blood Transfusion and the Indian Journal of Community Medicine) for English-language studies published between 2015 and 2026, using combinations of the terms "anaemia," "anemia," "pregnancy," "India," "NFHS," "iron deficiency," "Anaemia Mukt Bharat," and "haemoglobinopathy". We supplemented peer-reviewed literature with official government sources (Ministry of Health and Family Welfare communications, National Health Mission programme documents, and NFHS fact sheets) and reputable secondary reporting where primary government data were the underlying reference, given the significant role that recent (2026) programme and survey changes play in this argument. Seminal programme and policy documents predating the 2015 search window were additionally consulted to establish historical context. Given the narrative design, no formal quality appraisal or risk-of-bias tool was applied. Nationally representative sources were prioritised for prevalence estimates and peer-reviewed cohort or community-based studies for aetiological and determinant data.

The epidemiological picture: what we actually know, and what we do not

Any analysis of why India cannot fight anaemia must first confront an uncomfortable possibility: that the size of the problem, as officially measured, may partly reflect the measurement methodology rather than underlying biological prevalence.

NFHS-5 (2019-21) reported that 52.2% of pregnant women in India were anaemic, using the haemoglobin cut-off of <11.0 g/dL, as defined in WHO guidelines [1]. A 2025 reanalysis of NFHS trend data spanning 1998-2021, applying WHO's revised haemoglobin cut-offs, recalculated this figure downward to 47.1% [3,7]. The fact that a five-percentage point difference can arise entirely from a change in diagnostic cut-off, without any corresponding change in the study population, is notable. This methodological sensitivity may not be sufficiently emphasised in public reporting.

The second source of uncertainty concerns the blood sampling method itself. NFHS-4 and NFHS-5 estimated haemoglobin using point-of-care analysis of capillary blood obtained by finger prick in field settings. A methodological comparison study in rural Eastern Maharashtra, which collected both capillary and venous samples from the same cohort, found anaemia prevalence of 51.1% by capillary blood analysis versus 37.5% by venous cyanmethaemoglobin analysis in the first trimester, which is a 13.6-percentage point gap attributable to sampling method alone [8].

Against this background, the Ministry of Health and Family Welfare discontinued haemoglobin testing altogether from the sixth round of the NFHS (NFHS-6, fieldwork 2023-24, results released May 2026), citing accuracy concerns with capillary sampling. Broader NFHS-6 findings on maternal and reproductive health indicators, released alongside fact sheets, confirm the survey's continued scope in other domains, even without haemoglobin data [9]. Consequently, India currently lacks a nationally representative, contemporary estimate of the prevalence of anaemia during pregnancy.

Regional estimates continue to be generated. In a rural, predominantly tribal district of Jharkhand, anaemia prevalence among pregnant women was recorded at 92.8% using a digital haemoglobinometer between 2022 and 2024 [10]. Work across 10 districts of Gujarat documented substantial geographic disparities in rural, urban, and tribal populations within a single state [11]. At a teaching institution serving a rural population in Haryana, prevalence among women at term reached 98% over a 6-month period, with nearly 1 in 5 classified as severe or very severe [12]. These are local estimates, generated with inconsistent methods, and cannot be aggregated into a national picture.

District-level comparisons between NFHS-4 and NFHS-5 add further complications. Rather than showing a uniform decline, over 60% of India's 112 government-designated "Aspirational Districts" recorded an increase in the crude (unadjusted) prevalence of anaemia among all women of reproductive age between the two survey rounds. The crude prevalence within this group rose from 58.7% to 61.1%, and individual districts, such as Barpeta and Udalguri in Assam, saw increases exceeding 25 percentage points [13]. An adolescent-specific analysis of the same 2 survey rounds found that the prevalence of anaemia among girls aged 15-19 years increased from 54.0% to 59.2% nationally, with 21 of 28 states showing an increase [14]. The true population-level trajectory of anaemia in India remains uncertain. It may improve once corrected for cut-off and sampling method changes, or it may genuinely worsen in specific high-burden geographies and age groups. The current state of the surveillance infrastructure cannot confidently answer this question. A programme cannot be reliably judged to be failing or succeeding against a target that it can no longer measure. The sensitivity of the measured burden to sampling method and diagnostic cut-off is shown in Figure 1.

**Figure 1:**
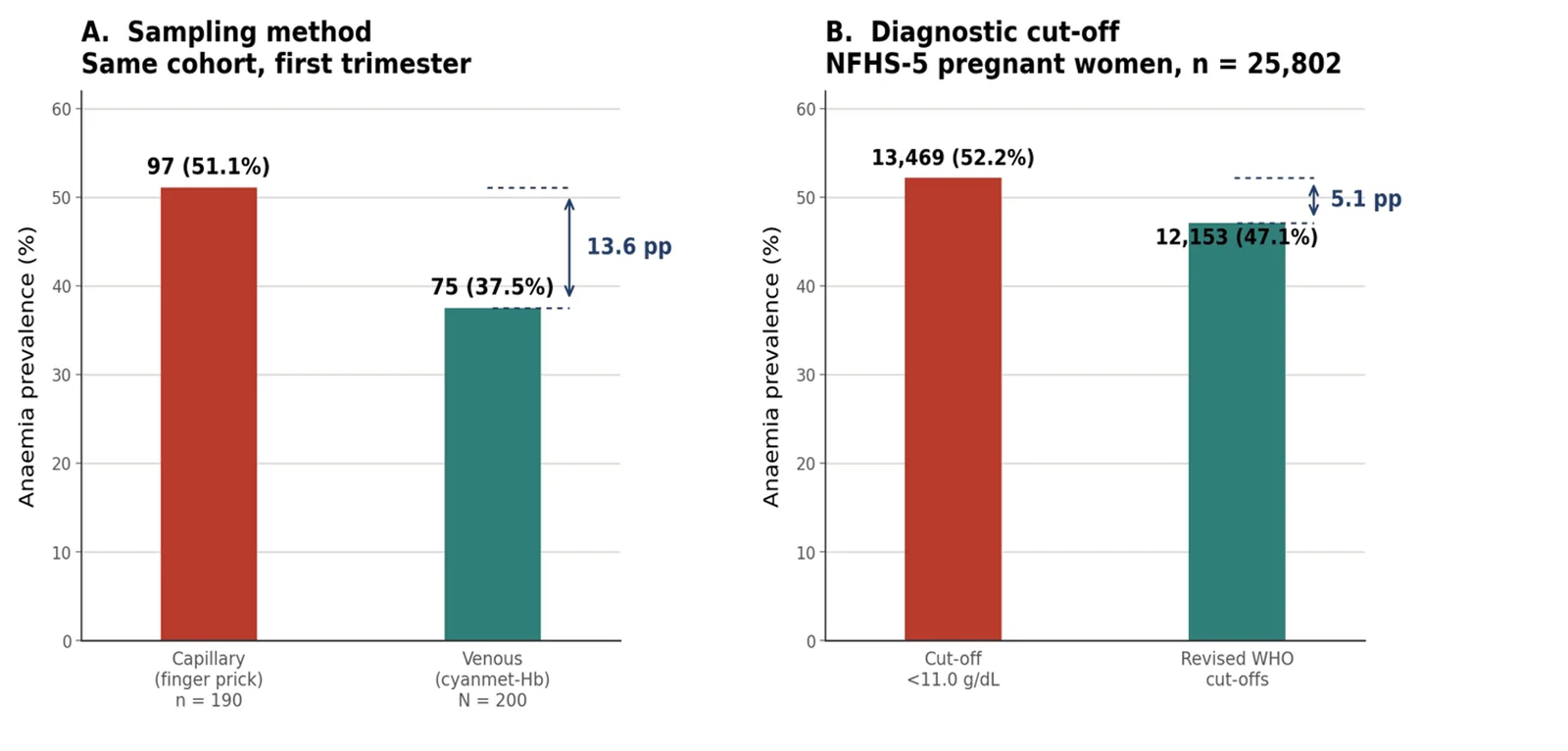
Measured anaemia prevalence varies by method, not biology Measured anaemia prevalence varies with method rather than with biology. (A) Paired capillary and venous samples from the same cohort in the first trimester in rural Eastern Maharashtra gave prevalence estimates of 51.1% and 37.5%, respectively, a difference of 13.6 percentage points attributable to sampling method alone [8]. (B) Applying revised WHO haemoglobin cut-offs to the identical NFHS-5 dataset moved the estimate from 52.2% to 47.1%, without any change in the study population [1,3,7]. pp, percentage points; NFHS, National Family Health Survey; cyanmet-Hb, cyanmethaemoglobin Image credit: Figure created by the authors using Python (Matplotlib) Data sources: panel A, Locks et al. [8]. Panel B is derived entirely from Pandey et al. [3], who reanalysed NFHS-5 microdata [1] for pregnant women (n = 25,775); the 52.2% estimate applies the previous haemoglobin cut-off and the 47.1% estimate the revised WHO trimester-specific cut-offs [7]. Counts in panel B are calculated from these published percentages and are not themselves published figures.

Why a biomedical framing alone is insufficient: anaemia is not synonymous with iron-deficiency anaemia

India's national anaemia programme has, since its inception, been built overwhelmingly around a single intervention logic: anaemia is caused by iron deficiency, and iron deficiency is corrected by supplementation. This biomedical framing conceptualises anaemia primarily as a nutrient-deficiency disorder amenable to pharmacological correction, rather than as a multifactorial condition shaped by aetiology, health-system capacity, and social context. This logic underlies iron-folic acid (IFA) tablet distribution, and in the Anaemia Mukt Bharat Abhiyaan's 2026 revision, an expanded role for intravenous iron preparations in moderate to severe cases [2]. However, evidence does not support treating anaemia and iron-deficiency anaemia as synonymous in the Indian context.

In the Eastern Maharashtra cohort discussed above, around 56% of women who were found to be anaemic by first-trimester venous blood testing showed biochemically confirmed iron deficiency (serum ferritin <15 μg/L). In other words, approximately 44% of anaemic women did not meet the study’s biochemical criterion for iron deficiency, while a further 19% of women were iron-deficient without being anaemic, indicating that iron and anaemia status diverge in both directions [8]. A separate hospital-based study from Kolkata specifically examined whether anaemia during pregnancy in eastern India is solely attributable to iron deficiency [15]. This aetiological picture is corroborated by a cross-sectional study conducted in rural Gujarat, which reported that 61.8% of anaemic mothers exhibited normal ferritin levels, indicating non-iron deficiency anaemia (NIDA) rather than iron deficiency [16]. A record review of 302 women attending a secondary-level facility in Tamil Nadu found that, despite 99.3% receiving iron and folic acid prophylaxis, 8.6% of the 174 women with anaemia required parenteral iron, and 16.7% required blood transfusion. As inclusion required at least six antenatal visits, these figures describe a well-attended cohort [17].

National-level data corroborate the scale of non-iron contributors. Folate deficiency has been estimated to affect 56.8% of women of reproductive age in India, and vitamin B12 deficiency affects 44.4% [6]. These figures reflect a widespread reliance on cereal-based diets with limited animal-source foods. Cohort-level evidence bears this out. In a tertiary-centre study of 172 anaemic antenatal women in Himachal Pradesh, 73.3% of those who were iron-deficient carried an additional folate or B12 deficiency, and 60.5% of the cohort had more than 1 nutritional deficiency [18]. Where such combined deficiencies predominate, dimorphic anaemia becomes the expected presentation, and it accounted for 48% of cases in an urban Delhi cohort, ahead of pure iron deficiency anaemia [19].

Two NIDA contributors deserve particular attention in the Indian context: beta-thalassaemia trait and sickle cell trait or disease. A 2024 systematic review and meta-analysis of Indian studies estimated the national prevalence of sickle cell disease at 1.17%, sickle cell trait at 5.9%, and HbS beta-thalassaemia at 0.37%, with higher rates among tribal populations and in states of Madhya Pradesh, Chhattisgarh, and Maharashtra [20]. The beta-thalassaemia trait carrier rate in India has been estimated to be nearly two to three times the global average at 1.5% [21]. They are misclassified and re-treated as iron deficient in routine antenatal screening that relies on haemoglobin concentration alone, without red cell indices or confirmatory testing. Chronic parasitic infections (hookworms) and other causes of anaemia of inflammation remain under-addressed [2,22].

Persistent anaemia is then interpreted as a failure of supplementation adherence. This is not a minor technical point: it means that even a hypothetically perfect IFA supply chain, with perfect compliance, would still fail to correct anaemia in a substantial proportion of pregnant women. 

Health-system and programme-design barriers

Distinguishing Iron Deficiency From Non-iron Deficiency Anaemia

Screening infrastructure capable of distinguishing iron deficiency from non-iron deficiency anaemia, including red cell indices, ferritin testing and haemoglobin electrophoresis, is largely unavailable below the district hospital level. Consequently, frontline antenatal care providers have little practical means of identifying approximately half of the anaemic women whose condition will not respond to iron alone [15,23]. This area needs financial and logistical support at grassroots level. Parenteral iron for moderate-to-severe cases has historically been concentrated at higher-tier facilities, leaving peripheral centres reliant on oral supplementation regardless of severity or underlying aetiology.

Logistic Management Problems

A mixed-methods investigation of the iron and folic acid supply chain in Bihar identified inaccurate demand forecasting, procurement delays, and breakdowns in facility-level storage and last-mile delivery as the principal bottlenecks [24]. These constraints persist more than a decade later. A 2026 implementation study in Haryana found that although all facilities reported holding IFA stock, six of eight had quantities insufficient for their registered pregnant population, with providers attributing this to programme activities commencing before supplies arrived [25].

Discordance Between Programme Strategy and Its Evaluation

The pace of strategic revision reflects the Government of India's ongoing efforts to modify the programme design in line with emerging requirements. The original National Nutritional Anaemia Prophylaxis Programme was superseded by Anaemia Mukt Bharat in 2018. This programme was structured around a "6×6×6" framework comprising six target beneficiary groups, six interventions, and six institutional mechanisms. It set an explicit target of reducing anaemia prevalence by three percentage points annually among children, adolescents, and women of reproductive age, from 2018 to 2022 [22]. Against this target, NFHS-5 data (2019-21) show national anaemia prevalence among women of reproductive age rising rather than falling, from 53% in NFHS-4 to 57% in NFHS-5 [1,26].

In June 2026, the Ministry of Health and Family Welfare released substantially revised operational guidelines, revising and renaming the programme as AMB Abhiyaan [2]. This revision expanded the framework to "7×7×7," adding low-birth-weight infants as a seventh beneficiary group alongside an "Eating Right" dietary diversity intervention. The operational model was also shifted from "T3" (Test, Treat, Talk) to "T4" (Test, Treat, Talk, Track), incorporating intensified haemoglobin testing, case-based tracking, and expanded access to intravenous iron.

It is worth noting the near-exact temporal coincidence between this redesign, which calls for intensified haemoglobin testing at the point of care, and NFHS-6's decision, made a month earlier, to abandon haemoglobin testing at the population survey level [9]. The health system is therefore being asked to test more intensively at the individual level, just as the principal tool for verifying whether that intensification works has been withdrawn. 

The Social Determinants Layer

The health system barriers described above affect women who, in a large proportion of cases, have already entered pregnancy with substantially depleted iron stores. This is the layer at which the "social issue" framing of India's anaemia burden is evident.

Age at marriage and first pregnancy is a central variable. NFHS-5 data show that approximately 56% of ever-married women aged 15-45 who were anaemic had been married before the age of 18. Multinomial regression analysis further found that early marriage, together with witnessing parental violence in childhood, was independently and significantly associated with anaemia status, even after adjusting for socioeconomic covariates [27,28]. NFHS-5's own reporting flagged a related concern. Both child marriage and adolescent pregnancy declined only marginally between NFHS-4 and NFHS-5, from 26.8% to 23.3% and from 8% to 6.7% respectively [1,26,29].

Adolescent pregnancy remains concentrated in specific populations and geographies. Rural prevalence (7.8%) is more than double the urban figure (3.8%), and regional clustering is pronounced across the eastern and northeastern states. Tripura recorded the highest burden, where more than one in five adolescents and one in four in rural areas reported pregnancy [30]. Adolescents who become pregnant are, biologically, still completing their linear growth. They are therefore competing with the fetus for iron.

A recent intersectional analysis of national survey data found that Scheduled Caste and Scheduled Tribe status independently predicts anaemia risk even after adjusting for household wealth, education, and residence, and that this caste-linked gap widened rather than narrowed between NFHS-4 and NFHS-5 [31]. The Aspirational Districts analysis found that women from Scheduled Tribe had 39% higher adjusted odds of anaemia, and women in the poorest wealth quintile had 27% higher odds, relative to their comparators, after adjusting for individual risk factors [13].

Maternal education was independently associated with anaemia status, with 13% lower adjusted odds among women with higher education and 8% lower odds among those with primary education [13]. This pattern is consistent with the broader literature on intra-household food distribution in South Asia, in which pregnant and lactating women frequently eat last and least within the household [32]. Non-consumption of iron-rich foods persists even when household income nominally permits better dietary options, owing to entrenched dietary norms and limited individual autonomy over food purchase and preparation [33].

Perception of anaemia further undermines compliance. Qualitative work in rural Odisha found risk perception to be low despite endemic prevalence, with fatigue normalised as an ordinary condition of motherhood rather than a sign of illness [34,35]. A widely reported belief that excess tablets would produce a large baby and a difficult delivery generated pressure, particularly from mothers-in-law, to take fewer than prescribed [34]. A 2025 synthesis across 23 countries confirms such determinants recur beyond India [36]. Where anaemia is understood in these terms, supplementation loses urgency regardless of how readily it is available.

Compliance with oral iron remains poor, undermined in part by side effects such as nausea, constipation, and stool discolouration. One cross-sectional study found that only 9.5% of pregnant women were consistent in their iron intake, and that 43.8% of women already diagnosed with anaemia were not taking iron supplements at all [27].

With respect to health-seeking behaviour and early booking, the same study also documented delayed care-seeking, with only 19% of women with anaemia having sought antenatal care by the second trimester and 22% presenting for the first time after 36 weeks of gestation [27]. Another study reported a mean consumption of only 27 IFA tablets across the course of pregnancy, far short of the 180 tablets recommended for a full course [37].

Parity and birth spacing add a further structural layer. A cross-sectional study of 904 pregnant women attending antenatal clinics in an urban setting in Uttar Pradesh, where the prevalence of anaemia was 59.6%, identified a short interpregnancy interval of under 24 months as a significant predictor of anaemia, alongside lower educational attainment and poor awareness of anaemia [38]. In a rural Delhi cohort, anaemia rose from 68.4% among primigravidae to 89.3% at gravida three or above (AOR 2.33) [39]. This pattern is not unique to India. A systematic review and meta-analysis of studies across the Asia-Pacific region found that a short inter-birth interval was associated with nearly threefold higher odds of anaemia relative to an optimal interval (OR 2.81, 95% CI 1.30-4.31) [40]. A rural cohort study reinforces that among the anaemic women in that sample, 71% had birth spacing of two years or less [41].

None of these determinants - age at marriage, adolescent pregnancy, intra-household food allocation, parity, and limited female autonomy over diet and health-seeking - is directly addressed by any iteration of Anaemia Mukt Bharat's core intervention package. Notably, they are the variables that show the strongest statistical association with anaemia status in the studies reviewed here, often larger in magnitude than variables directly targeted by supplementation programming.

Structural and governance barriers

Cross-sectoral governance constitutes a major layer positioned above both the health system and individual household behaviour. The non-nutritional contributors to anaemia include: i) poor sanitation, ii) hookworm infestation, iii) malaria in endemic regions, and iv) a higher concentration of haemoglobinopathies within tribal populations. The contribution of helminthic infection is not merely theoretical. In a rural Delhi cohort, a history of worm infestation was independently associated with anaemia (AOR 4.82), with 93.3% of women reporting worms in stools found to be anaemic. The authors observed that routine deworming remains underused within antenatal care despite its inclusion in programme guidance [39].

These contributors fall under the remit of ministries and missions such as drinking water and sanitation, vector-borne disease control, and tribal affairs. Each of these operates largely independently of the health ministry's nutrition programming, despite AMB's stated "Whole of Government" ambition [2]. The 2026 Anaemia Mukt Bharat Abhiyaan guidelines explicitly invoke inter-ministerial convergence and community mobilisation, "Jan Bhagidari" and "Jan Chetna,” as governance principles.

State-level implementation variance further complicates any uniform national narrative. The Aspirational Districts data reveal considerable heterogeneity across districts. This suggests that state and district-level health system capacity, governance quality, and local social conditions interact with programme delivery in ways that a centrally designed intervention package cannot fully anticipate or correct.

Synthesis: a structural failure, not a deficiency of effort

Bringing the four domains together, India's persistent anaemia burden cannot be blamed on any single actor's lack of effort. Although the programme includes multiple interventions, its dominant operational emphasis remains biomedical and supplementation-centred, while several social, environmental, and aetiological determinants remain incompletely addressed. Measurement uncertainty further leaves it unclear whether even the biomedical layer is being resolved. Table 1 sets out each layer with its supporting evidence, the conclusion it warrants, and the corresponding recommendation.

**Table 1:** Determinant layers of persistent anaemia in pregnancy in India: evidence base, conclusions and recommendations AMB, Anaemia Mukt Bharat; IFA, iron and folic acid; NFHS, National Family Health Survey; WHO, World Health Organization

Sl. no	Determinant layer	Key references	Conclusion	Recommendation
1	Epidemiological (measurement)	[1,3,7-14]	Prevalence is method-sensitive: capillary sampling gave 51.1% against 37.5% by venous analysis in the same cohort. Revised WHO cut-offs alone moved the NFHS-5 estimate from 52.2% to 47.1%. Haemoglobin testing was then removed from NFHS-6, leaving no contemporary national estimate. Regional figures (92.8% in Jharkhand, 98% at term in Haryana) use inconsistent methods. Over 60% of the 112 Aspirational Districts recorded a rise, and adolescent prevalence rose from 54.0% to 59.2%.	Reinstate haemoglobin measurement in future NFHS rounds using validated venous or dual-method protocols. Standardise the cut-off and sampling method across national and state surveys so trends are comparable. Report method and cut-off alongside every published prevalence figure.
2	Biomedical (aetiology)	[2,6,8,15-22]	Anaemia is not synonymous with iron deficiency anaemia. About 44% of anaemic women had normal ferritin in Maharashtra and 61.8% in rural Gujarat. A further 19% were iron deficient without being anaemic. Folate deficiency affects an estimated 56.8% and vitamin B12 deficiency 44.4% of women of reproductive age. In one anaemic antenatal cohort, 60.5% had more than one nutritional deficiency. Sickle cell trait (5.9%) and beta-thalassaemia trait (1.5%) are misclassified where screening relies on haemoglobin alone.	Incorporate aetiology-differentiated screening into routine antenatal care rather than confining it to referral facilities. Provide red cell indices alongside haemoglobin at frontline level. Establish referral pathways for confirmatory ferritin and electrophoresis instead of escalating iron dose. Address deworming and infection-related anaemia in endemic districts.
3	Health system (programme design)	[1,2,9,15,22-26]	Red cell indices, ferritin and electrophoresis are largely unavailable below district hospital level. Frontline providers therefore cannot identify the roughly half of anaemic women who will not respond to iron. Supply-chain bottlenecks trace to forecasting, procurement and last-mile delivery, and persist more than a decade later. The programme has been redesigned twice in eight years while prevalence among women of reproductive age rose from 53% to 57%. Intensified individual-level testing was mandated in the same period that population-level verification was withdrawn.	Strengthen demand forecasting, procurement and last-mile logistics before any further strategic redesign. Extend point-of-care diagnostic capability and parenteral iron access to peripheral facilities. Evaluate each programme iteration against a fixed measurement standard before the next revision.
4	Social (determinants)	[13,27-41]	Around 56% of anaemic ever-married women were married before 18, while child marriage fell only from 26.8% to 23.3%. Pregnant adolescents are still growing and compete with the fetus for iron. Scheduled Tribe women had 39% higher adjusted odds of anaemia, and the poorest quintile 27%. Women eat last and least within the household, and fatigue is normalised as a condition of motherhood. Only 9.5% took iron consistently, and mean consumption was 27 of 180 tablets. 22% first presented for antenatal care after 36 weeks. Interpregnancy interval under 24 months independently predicted anaemia.	Shift the intervention window upstream through adolescent nutrition programming, school-based supplementation and delayed age at marriage. Design demand-side interventions engaging husbands and mothers-in-law. Prioritise high-burden districts and tribal populations with targeted rather than uniform packages. Integrate anaemia counselling with birth spacing services.
5	Governance (cross-cutting)	[2,5,13,22,39,42-46]	Sanitation, hookworm, malaria and haemoglobinopathy sit with ministries operating independently of health, despite a stated Whole of Government ambition. Heterogeneity across Aspirational Districts indicates that local capacity and governance quality shape delivery in ways a centrally designed package cannot anticipate. Sri Lanka reaches 29.1% through lifecycle-wide delivery, its residual burden dominated by haemoglobinopathy and B12 deficiency. The Philippines mandates staple fortification; Bangladesh pairs supplementation with women's self-help groups.	Replace aspirational convergence language with enforceable cross-sectoral accountability. Track sanitation coverage, girls' school retention and age at marriage on the same dashboard as haemoglobin testing and IFA distribution. Consider mandatory staple fortification, which requires neither clinic attendance nor behaviour change. Fund the 'Eating Right' and 'Jan Bhagidari' components as substantive interventions.

This pattern is worth setting against countries with lower measured prevalence. Sri Lanka offers the most directly comparable case. Its prevalence of anaemia in pregnancy stands at 29.1%, well below India's, achieved through a public health infrastructure delivering interventions across the reproductive lifecycle rather than at antenatal registration alone [42]. The residual burden is itself revealing: a first-trimester cohort found that although iron deficiency predominated, minor haemoglobinopathies and B12 deficiency contributed substantially, prompting calls for haemoglobinopathy screening and multiple micronutrient supplementation [43]. Stronger delivery lowers prevalence, but the fraction iron cannot address becomes the limiting factor.

The Philippines has mandated fortification of rice, wheat flour, and cooking oil with iron and other micronutrients, an intervention requiring neither behaviour change nor clinic attendance, with pilot areas showing reductions in anaemia prevalence of 10 to 15% within two years [44]. In Bangladesh, iron supplementation has been combined with women's self-help groups that grow iron-rich vegetables and involve husbands and mothers-in-law in supporting women's diets, tackling the household food-sharing problem seen in India [45]. A review of anaemia control programming in low- and middle-income countries identified the strongest enabling factors as socio-ecological rather than supply-side: maternal literacy, partner support, community ownership, and use of trusted existing infrastructure, such as schools and antenatal clinics, to deliver supplements while shifting household decision-making norms [46].

India's own programme history reflects some awareness of this logic. The "Eating Right" and "Jan Bhagidari" components noted earlier point towards upstream, non-clinical intervention. Much will depend on whether these components are funded and delivered seriously.

Recommendations for a reoriented approach

Four shifts follow from this analysis in four domains: surveillance, screening, timing, and governance.

First, surveillance must be restored on a methodologically defensible basis, reinstating haemoglobin measurement in future NFHS rounds using validated venous or dual method protocols.

Second, aetiology-differentiated screening should be incorporated into routine antenatal care rather than being confined to referral facilities. Even basic red cell indices alongside haemoglobin would allow frontline workers to flag probable non-iron deficiency, rather than escalating iron dose in every case.

Third, interventions must begin well before antenatal registration through adolescent nutrition programming, delayed marriage, and school-based supplementation, since the iron deficit is already established by the time a woman conceives.

Fourth, governance requires enforceable accountability, not aspirational language. Sanitation coverage, girls' school retention, and age at marriage should be tracked alongside haemoglobin testing and IFA distribution on a single dashboard so that the evaluation extends beyond what the health ministry alone controls.

Limitations of this review

This is a narrative, not a systematic review, and the included studies varied considerably in geography, sample size, and haemoglobin measurement method. Several key data points, including Aspirational Districts and adolescent trend analyses, were derived from comparisons between NFHS-4 and NFHS-5 and therefore predate NFHS-6. Because NFHS-6 did not collect haemoglobin data, no directly comparable national updates exist at the time of this writing. Comparative international examples were included illustratively rather than through systematic cross-country evaluation and may not be fully generalisable to India's scale and federal structure.

## Conclusions

Anaemia in pregnancy in India sits at the intersection of measurement uncertainty, biological heterogeneity, programme design, and deeply rooted social structure. Each layer has, at some point, been treated as the whole problem, and most often, anaemia has simply been equated with iron deficiency. The persistence of a high prevalence across four decades of programming and the fact that the national programme has now required two major structural overhauls suggest that a single-lever approach has reached the limit of what it can deliver.

A strategy that restores reliable surveillance, differentiates aetiology at the point of care, intervenes across the life course rather than only during pregnancy, and treats social determinants as core programme targets rather than contextual background offers a more plausible way forward.

## References

[1] (2026). International Institute for Population Sciences (IIPS). National Family Health Survey (NFHS-5), 2019-21. https://dhsprogram.com/pubs/pdf/FR375/FR375.pdf.

[2] (2026). Ministry of Health and Family Welfare, Government of India. Anemia Mukt Bharat (AMB) Abhiyaan. Revised operational guidelines. Anemia Mukt Bharat (AMB) Abhiyaan: Revised Operational Guidelines. New Delhi: MoHFW.

[3] Pandey AK, Gautam D, M BT, Neogi SB (2025). Trends in anemia prevalence among Indian women using revised WHO hemoglobin cutoffs: insights from repeated cross-sectional surveys (1998-2019). Anemia.

[4] Benavente MT, Geifman N, Bath S, Ahankari A (2025). Maternal anemia and offspring outcomes in India: a scoping review. Anemia.

[5] (2026). Anemia Mukt Bharat. Operational guidelines. https://www.nhm.gov.in/New-Update-2025-26/Nutrition/AMB-guidelines/AMB-Abhiyaan-Guidelines.pdf.

[6] Neogi SB, Babre A, Varghese M, Hallen JB (2022). Improving the approach to assess impact of anaemia control programs during pregnancy in India: a critical analysis. BMC Pregnancy Childbirth.

[7] (2026). World Health Organization. Guideline on haemoglobin cutoffs to define anaemia in individuals and populations. https://www.who.int/publications/i/item/9789240088542.

[8] Locks LM, Bhaise S, Dhurde V (2024). The prevalence of anemia during pregnancy and its correlates vary by trimester and hemoglobin assessment method in Eastern Maharashtra, India. Matern Child Nutr.

[9] (2026). National Family Health Survey (NFHS-6), 2023-24: India and State/UT fact sheets. https://www.nfhsiips.in/nfhsuser/release-details.php.

[10] Bhushan A, Sagar V, Kujur A (2024). Burden and predictors of anemia among pregnant and lactating females in a rural area in India with a high tribal population. Cureus.

[11] Agarwal A, Bakshi H, Kadri AM (2025). Assessing anemia burden and multifactorial contributors among pregnant women in Gujarat: a cross-sectional study integrating biochemical, nutritional, and geographic disparities. Front Glob Womens Health.

[12] Mangla M, Singla D (2016). Prevalence of anaemia among pregnant women in rural India: a longitudinal observational study. Int J Reprod Contracept Obstet Gynecol.

[13] Let S, Tiwari S, Singh A, Chakrabarty M (2024). Prevalence and determinants of anaemia among women of reproductive age in Aspirational Districts of India: an analysis of NFHS 4 and NFHS 5 data. BMC Public Health.

[14] Ibirogba D, Menon VB, Olickal JJ, Thankappan KR (2024). Trends in prevalence and predictors of anemia in adolescents between the ages of 15 and 19 years in India and its states: evidence from the National Family Health Survey 2015-16 and 2019-21. Cureus.

[15] Ghosh M, Bhattacharyya M (2026). Anemia in pregnancy - is it all iron deficiency?. Indian J Hematol Blood Transfus.

[16] Saha S, Puwar T, Shah K, Pandya A, Wanjari MB, Saxena D (2022). Non-iron deficiency anemia in rural Indian women: a cross-sectional study. Cureus.

[17] Biradar B, Arasu S, Ramesh N (2023). Anemia burden in pregnancy and birth outcomes among women receiving antenatal care services from a secondary level hospital in South India: a record review. J Family Med Prim Care.

[18] Kaushal S, Priya T, Thakur S, Marwaha P, Kaur H (2022). The etiology of anemia among pregnant women in the hill state of Himachal Pradesh in North India: a cross-sectional study. Cureus.

[19] Eram A, Trivedi SS, Kumar M, Naqvi Sayyed EH (2017). A cross sectional study of prevalence of ‘anemia in pregnancy’ and its outcome in urban population of New Delhi. JMSCR.

[20] Rao P, Raj EA, Natesan S, Gudi N (2024). Prevalence of sickle cell disease, sickle cell trait and HBS-beta-thalassemia in India: a systematic review and meta-analysis. Clin Epidemiol Glob Health.

[21] Colah R, Italia K, Gorakshakar A (2017). Burden of thalassemia in India: the road map for control. Pediatr Hematol Oncol J.

[22] (2026). National Health Mission, Government of India. Anaemia Mukt Bharat. Programme overview. https://nhm.gov.in/index1.php?lang=1&level=3&sublinkid=1448&lid=797.

[23] Lauer JM, Bhaise S, Dhurde V (2024). Maternal anemia during pregnancy and infant birth outcomes: a prospective cohort study in eastern Maharashtra, India. Curr Dev Nutr.

[24] Wendt AS, Stephenson R, Young MF (2018). Identifying bottlenecks in the iron and folic acid supply chain in Bihar, India: a mixed-methods study. BMC Health Serv Res.

[25] Gadapani Pathak B, Vats P, Jadaun A, Garg N, Mazumder S (2026). Implementation challenges of India's national anaemia reduction program among pregnant women: insights from mixed-methods implementation research. Front Glob Womens Health.

[26] (2026). International Institute for Population Sciences (IIPS) and ICF. National Family Health Survey NFHS-4, 2015-16. https://dhsprogram.com/publications/publication-fr339-dhs-final-reports.cfm.

[27] Mishra A, Marwah S, Divedi P, Dewan R, Ahluwalia H (2021). A cross-sectional study of barriers in prevention of anemia in pregnancy. Cureus.

[28] Mishra T (2026). The effect of early marriage and witnessing parental violence in childhood on anemia status of Women in India: some insights from NFHS 5. SN Soc Sci.

[29] (2026). United Nations Population Fund (UNFPA) India. Child marriage in India: key insights from the NFHS-5. https://india.unfpa.org/en/publications/analytical-paper-series-1-child-marriage-india-key-insights-nfhs-5-2019-21.

[30] Halder P, Gloria A, Prasad IS (2026). Adolescent pregnancy in India: multilevel insights into distribution, determinants and socioeconomic inequalities. BMJ Public Health.

[31] Das B, Adhikary M, Singha SR, Parmar D (2024). Who is anaemic in India? Intersections of class, caste, and gender. J Biosoc Sci.

[32] Lentz EC, Narayanan S, De A (2019). Last and least: findings on intrahousehold undernutrition from participatory research in South Asia. Soc Sci Med.

[33] Harris-Fry H, Shrestha N, Costello A, Saville NM (2017). Determinants of intra-household food allocation between adults in South Asia - a systematic review. Int J Equity Health.

[34] Sedlander E, Long MW, Mohanty S, Munjral A, Bingenheimer JB, Yilma H, Rimal RN (2020). Moving beyond individual barriers and identifying multi-level strategies to reduce anemia in Odisha India. BMC Public Health.

[35] Sedlander E, Talegawkar S, Ganjoo R, Ladwa C, DiPietro L, Aluc A, Rimal RN (2021). How gender norms affect anemia in select villages in rural Odisha, India: a qualitative study. Nutrition.

[36] Verbunt E, Corona MV, Tunçalp Ö, Rogers LM, Prang KH, Vaughan C, Bohren MA (2025). Perceptions and experiences of the prevention, testing, and treatment of anaemia in pregnant women: a qualitative evidence synthesis. PLOS Glob Public Health.

[37] Chakrabarti S, George N, Majumder M, Raykar N, Scott S (2018). Identifying sociodemographic, programmatic and dietary drivers of anaemia reduction in pregnant Indian women over 10 years. Public Health Nutr.

[38] Kumar P, Ahmad S, Davey A, Saxena K (2026). Prevalence and predictors of anaemia in pregnancy: the role of birth spacing, educational status and awareness levels in Meerut City. Int J Community Med Public Health.

[39] Jegadeesan D, Rajendran V, Rajamanickam S (2026). Prevalence of anemia and associated factors among pregnant women attending a rural primary health care facility in Delhi. Cureus.

[40] Feyissa TR, Chojenta C, Hassen TA, Beyene T, Khan MN, Bagade T, Harris ML (2025). Short birth/pregnancy interval and its association with adverse maternal outcomes in Asia Pacific region: a systematic review and meta-analysis. Midwifery.

[41] Singal N, Setia G, Taneja BK, Singal KK (2018). Factors associated with maternal anaemia among pregnant women in rural India. Bangladesh J Med Sci.

[42] (2026). Family Health Bureau Sri Lanka. Annual report of the family health bureau 2018. Bureau.

[43] Amarasinghe GS, Agampodi TC, Mendis V, Malawanage K, Kappagoda C, Agampodi SB (2022). Prevalence and aetiologies of anaemia among first trimester pregnant women in Sri Lanka; the need for revisiting the current control strategies. BMC Pregnancy Childbirth.

[44] (2026). Philippine Statistics Authority (PSA). 2022 Philippine National Demographic and Health Survey (NDHS). Final report. Quezon City (Philippines): PSA.

[45] (2026). National Institute of Population Research and Training (NIPORT) and ICF. Bangladesh Demographic and Health Survey 2022: final report. Dhaka, Bangladesh, and Rockville, Maryland, USA: NIPORT and ICF. https://dhsprogram.com/publications/publication-fr386-dhs-final-reports.cfm.

[46] Ge S, Ali S, Haldane V, Bekdache C, Tang GH, Sholzberg M (2025). An approach to hemequity: identifying the barriers and facilitators of iron deficiency reduction strategies in low- to middle-income countries. Br J Haematol.

